# Managing vulnerability: cross-sector reflections on the ethics and equity implications of AI use among young adults with chronic conditions

**DOI:** 10.3389/fdgth.2026.1891178

**Published:** 2026-08-28

**Authors:** MacKenzie Isaac, Sneha Dave, Terry Adirim

**Affiliations:** 1 Nuffield Department of Population Health, Medical Sciences Division, University of Oxford, Oxford, United Kingdom; 2 Generation Patient, Indianapolis, IN, United States; 3 Departments of Pediatrics and Preventative Medicine and Statistics, Uniformed Services University of the Health Sciences, Bethesda, MD, United States

**Keywords:** AI ethics and governance, chronic illness management, design justice, health and digital literacy, health equity, patient-centered design, responsible technology development, young adult patients

## Abstract

Generation Patient, a global advocacy organization led by young adults with chronic and rare medical conditions, convened a Roundtable in December 2025 to explore the potential and realized impacts of increased AI use in patient care. Across two hours, fifteen participants with a blend of lived, scholarly, and professional expertise engaged in a semi-structured focus group on the benefits and drawbacks of using AI to meet their healthcare needs within and beyond the clinic. Reflexive thematic analysis of the Roundtable transcript shed light on AI's promise to ease the administrative and emotional burden for young adults with complex conditions transitioning into adult care. However, increased use of AI for clinical administrative support raises concerns about patient data protection, stewardship, and equity. The Roundtable also extensively discussed the “double edged sword” of generative and conversational AI's tendency to affirm users' inputs, particularly in the case of mental health companion chatbots. Committed to translating these insights into actionable steps, participants recommended that AI developers and distributors adopt a DesignJustice approach—modeled by the Roundtable itself—to collaboratively build AI tools with end users and health equity experts who can support the creation of ethical standards, marketing strategies, and impact evaluations that focus on reducing harm and maximizing social good. At a policy level, broadening definitions of what constitutes a “medical device” to any AI tool with the capability of processing health-related data will increase the scope of the federal government's obligation and ability to protect patients.

## Introduction

Artificial intelligence (AI) holds enormous promise for transforming healthcare delivery, streamlining diagnostics, personalizing treatment, reducing administrative burden, and accelerating drug discovery at speeds previously unimaginable. Yet promise alone is insufficient justification for uncritical adoption, particularly when the populations standing to benefit most are also among the most vulnerable to harm. For young adults managing chronic and rare conditions, AI-enabled care carries a dual potential: to meaningfully extend their autonomy and quality of life, or to quietly reproduce the very inequities such as diagnostic delays, data exclusion, and institutional distrust, that already shape their healthcare experiences.

Working towards the former while establishing guardrails against the latter demands more than technical rigor. It demands the active, systematic involvement of patients themselves in the design, selection, and implementation of AI powered tools. This is not merely an ethical aspiration; it is a practical imperative. AI systems trained on historically non-representative clinical data risk encoding the biases of the research ecosystems that produced them, automating inequity at scale rather than dismantling it. Without patient partnerships that bring lived expertise into the development pipeline, healthcare AI is liable to optimize for the populations already well-served by existing systems, leaving those with the greatest unmet needs further behind.

This commentary draws on insights from a December 2025 Roundtable on Young Adults with Chronic Medical Conditions, convened by Generation Patient, to examine the intersecting ethics and equity implications of AI in young adult patient care. Using AI mental health companion chatbots as a topical case, we propose multilevel safeguards to minimize AI-exacerbated health inequities and advocate for a Design Justice model (1) requiring the co-design of AI innovations and user guidelines with young adult patients and others with lived expertise at the table from the start, not as an afterthought.

## Convening details and methodology

On December 11, 2025, Generation Patient convened a group of stakeholders ranging from young adult patients and patient advocates to clinicians, bioethicists, health systems researchers, and philanthropists to participate in an AI-focused Roundtable on Young Adults with Chronic and Medical Conditions. All Roundtable participants and their scopes of experience and expertise can be found in Table 1. The primary author of this paper (MI), a health education specialist and young adult patient living with a chronic illness, participated in the roundtable as both a presenter and discussant. With the informed consent of all co-discussants, she and the second author (Generation Patient's Executive Director, SD) obtained a written transcript of the convening and took a reflexive thematic analysis approach to consolidate key takeaways and calls to action.

**Table 1:** Overview of roundtable participant demographics and expertise.

Discussant Number	Initials	Identity Description/Positionality Within Group	YA Patient with Lived Experience?
1	JB	Patient advocate and parent of a YA patient with multiple chronic conditions	No
2	AR	Patient and family advocate; student researcher who also works in tech/regularly interacts with AI in a professional capacity	Yes
3	PM	Health Policy Advocate/thought leader working for Generation Patient	Yes
4	AD	Non-profit leader of grassroots, patient-led advocacy organization	Former (current adult)
5	TA^a^	Physician and health policy expert with extensive federal government experience	No
6	YP	Young adult advocate and university student navigating the care transition from home to college	Yes
7	SD^a^	Non-profit leader of YA patient advocacy organization and health policy expert; Roundtable Convener	Yes
8	RB	Pediatric Fellow (neonatal care) who uses tech-intensive care models directly in professional practice	No
9	CS	Pediatrician and consultant/grassroots advocate for multiple YA patient and family advocacy organizations	Yes
10	AM	Postdoctoral Research Fellow studying responsible and ethical AI	No
11	RL	Patient advocate and subject matter expert on the socioeconomic stressors of healthcare systems navigation	Yes
12	SP	Health Policy academic focused on biotechnology and (comparative global) bioethics	No
13	CF	Mental health therapist and patient advocate who co-facilitates peer support groups for Generation Patient	Yes
14	SS	Medical researcher and community organizer for responsible and ethical medical technology use	No
15	MI^a^	Bioethics and health equity doctoral researcher who co-facilitates peer support groups for Generation Patient	Yes

a Denotes co-author of this proceedings paper.

Given our own identities as patients and patient advocates, the authors agreed that reflexive thematic analysis was the most appropriate interpretive framework for these Roundtable proceedings. Although our analysis and subsequent calls to action are drawn from a thoughtfully curated group of diverse stakeholders, we recognize that our blends of lived and professional expertise inevitably shape how we extract, interpret, summarize, and highlight certain data. The six-phase method described by Virginia Braun and Victoria Clarke (2) enabled us to document our biases while providing as comprehensive an overview of the Roundtable as possible:

1. The first author (MI) established familiarity with the data by reading the Roundtable transcript in full on two occasions. During the second reading, she compiled a reflexive journal (3), recording by hand the strong emotions, assumptions, dissenting opinions, and biases that surfaced at points of contention in the conversation. She further documented the ways in which her own perspectives on AI shifted upon revisiting the experiences and opinions of other participants.
2. MI reviewed the transcript a third time, coding the data openly on a line-by-line basis and attending to the most compelling arguments presented by participants. Approximately halfway through open coding, clear patterns in opinion stratified by age and profession become apparent.
3. These patterns were consolidated into dozens of candidate themes. We had anticipated that themes would fall along a spectrum of “positive versus negative sentiment”. Because this binary proved to be too reductive to accommodate the layered and balanced perspectives shared among participants, the authors adopted a strictly inductive approach to identifying key takeaways.
4. A consensus meeting among the authors yielded a shortlist of three themes, each of which was assigned representative quotations, with priority given to the perspectives of young adult patient participants in keeping with Generation Patient's advocacy priorities. All participants provided informed consent prior to the Roundtable to be quoted in this proceedings paper and in other community-facing organizational outputs concerning the role of AI in patient support.
5. After collaboratively assigning shorthand names to each shortlisted theme, the authors reordered the presentation of themes to construct as compelling, balanced, rigorous, and actionable narrative as possible.
6. Once MI had outlined the data narrative and SD and TA had validated it, all three authors worked asynchronously to prepare these reflections.

Convening a Roundtable of fifteen emerging and established thought leaders carries a methodological limitation. Given the numerous competing demands on participants' time, the standard practice of circulating the final codebook to all participants to confirm that each felt accurately and fairly represented in the analysis, a process known as member checking, proved logistically challenging. Furthermore, given the complexity and emotional weight that frequently accompany living with a chronic condition in young adulthood, revisiting the conversation in written form may have exacted an emotional toll on participants who extended the limits of their vulnerability during the Roundtable itself (4). Whether the rigor and credibility conferred by member checking outweighs the attendant risks to participants, whose emotional safety remains a paramount priority, warrants careful ethical consideration.

## Results

### AI as a tool to reduce burden

Roundtable participants broadly characterized AI as a promising means of alleviating administrative, cognitive, and emotional burden for young adults and their clinicians, rather than as a substitute for relationship-centered care. Efficiency gains were viewed as most valuable when they protected time and energy for meaningful human interaction. As one healthcare provider attending the roundtable noted, “I see a huge potential benefit in AI automating certain routine or mundane tasks and allowing clinicians more time for relationship-driven care”.

Participants supported this insight, sharing that they faced substantial paperwork and documentation demands that often needed to be repeated with each new care specialist. AI-enabled automation of routine tasks was viewed as a way to reduce this load for both patients and clinicians, freeing up capacity to discuss non-administrative matters during clinical encounters that are often already time-constrained. Insurance navigation and multidisciplinary care coordination emerged as additional domains in which AI could play a constructive role. Young adults described challenges in understanding benefits, responding to denials, and synchronizing complex care. They envisioned tools capable of translating insurance language, tracking deadlines, and supporting preparation for interactions with insurers and care teams.

Participants further explored the AI's potential to facilitate the transition from pediatric to adult care. AI tools, as participants suggested, could scaffold the assumption of self-management responsibilities by clarifying shifts in insurance coverage, summarizing care plans, streamlining patient data sharing between pediatric and new adult providers, and supporting communication between young adults and their care networks. For young adults who have faced medical discrimination, symptom dismissal, or protracted diagnostic processes, recounting their health histories to a new slate of providers can feel especially daunting and tedious (5). These patients may especially benefit from secure data-sharing systems that pre-upload their medical records for new specialists. In this context, AI could function as a mechanism of both efficiency and emotional protection.

### Areas of concern and governance gaps

Participants identified a well-documented concern that, in the absence of robust governance and participatory design, AI systems may exacerbate health inequities, undermine trust, and even introduce novel forms of harm (6). Several noted that many commercial solutions appeared misaligned with real-world workflows and needs, underscoring the importance of actively involving young adults and clinicians throughout development processes.

Participants also expressed unease with systems optimized for engagement or agreeableness, citing the sycophantic tone of voice and overly reassuring responses common to conversational AI (7). Mitigating this unease will involve striking a delicate balance between user validation and the sharing of accurate, actionable information, offering up another opportunity for patients and their care networks to be directly involved in the design, deployment, oversight, and impact evaluation of these emerging technologies.

Participants further expressed concerns about data privacy. They questioned who could access data shared with AI tools and how such information might be monetized or otherwise used to shape corporate and governmental decision-making. Participants noted that patients' fears of data misuse could lead them to self-censor when communicating with their clinicians, resulting in less timely or comprehensive care. Drawing from personal experiences, participants deemed consent and transparency practices around AI use in clinical encounters, including the use of AI scribes to summarize appointments, as hasty and insufficient. Many were uncertain when AI tools were in use, how data were processed, or whether they could decline participation, especially when refusal was perceived as potentially straining the clinical relationship. One person shared, “If you say no [to AI use] you’re getting on your doctor's nerves…I don't feel like I’m in a situation where I can say no”, a sentiment that resonated widely among patient attendees. They advocated for explicit disclosure of AI use and clear labeling of AI-generated or AI-edited clinical documentation as a minimum standard.

Participants additionally highlighted gaps in digital literacy among both patients and providers (8), which impede informed decision-making about AI. They expressed particular concern about the use of AI in high-stakes administrative and clinical determinations, such as prior authorization and medical necessity, noting that without proper training and oversight, users risk obscuring accountability behind ostensibly “objective” systems.

A critical aspect of digital literacy is understanding the relationship between data stewardship and data representation. AI-generated insights are bound by the quality of the data on which the tools are trained (9), which may reflect value systems, frameworks, or methodologies that have since been proven outdated or harmful. Where the history of clinical research participation for marginalized communities is fraught with structural exclusion and exploitation, particularly in the context of chronic and rare disease management (10), AI may echo these injustices and, in effect, automate bias.

### Topical example: AI companion tools and chatbots as potential medical devices

The increasing popularity of AI mental health companion tools, particularly chatbots, was a focal point of discussion and collective concern. Participants observed that these tools are widely used by young adults outside formal care yet often lack rigorous evaluation and regulatory oversight commensurate with their influence on mental health support and crisis responses (11).

One major challenge raised by AI chatbots is that they are not regulated as medical devices when marketed as consumer tools, even though many of them are used as such (11). Instead, they are routinely brought to market as “wellness” tools, and this framing loophole, ethically precarious as it is, allows many chatbot developers to evade liability when users experience adverse medical outcomes tied to their products.

Participants underscored the importance of advocacy efforts to classify such tools as medical devices in ensuring basic safety, transparency, and accountability. This is particularly demanding for young adults who may turn to these tools in the midst of medical distress or when physical, financial, or stigma-related barriers limit access to human-facilitated care. The risk that AI will reinforce or normalize harmful cognitions due to its sycophantic tone, particularly when algorithms prioritize responsiveness over clinical appropriateness, is especially acute in the mental health chatbot context.

It may be argued that classifying conversational AI and low-risk wellness tools as medical devices will initiate a “slippery slope”, imposing unnecessary requirements on products never designed to offer professional-grade health advice. A risk-based approach to patient advocacy, in preference to a reactive, compliance-based one, requires careful examination of all plausible ways in which people may interact with a product, together with proactive measurement and monitoring of the gap between intended and actual use. Conversational AI tools commonly publish terms and conditions disclosures that they are meant for informational purposes only, and that they may even be prone to hallucinating sources and facts (12). Such disclosures may do little to dissuade a young adult in emotional distress or chronic pain from seeking time-sensitive medical advice when external supports are not immediately accessible.

Ramaswamy et al. (13) subjected ChatGPT to a structured stress test comprising several clinician-approved medical vignettes and found that the model undertriaged 52% of cases constituting a medical emergency. In a separate study, Bean et al. (14) asked lay users, defined as individuals without medical training, to present ten medical scenarios to large language models in their own words. Participants correctly identified the health conditions underlying the scenarios in 34.5% of cases and identified the most appropriate course of treatment or intervention in 44.2% of cases.

In contrast to the performance observed by Ramaswamy et al., the models studied by Bean and colleagues proved capable of identifying and triaging conditions when tested in isolation. The introduction of human interaction, however, brought with it substantial subjectivity, variable interpretation, bias, and unintentional omission, rendering the models less effective as a healthcare support tool under real-world conditions. Distinguishing whether an individual required emergency care, should contact a helpline, or may reasonably continue monitoring their symptoms at home can carry life or death consequences. Considered together, these two studies demonstrate that such distinctions depend upon a combination of high model accuracy and high population health literacy that has not yet been achieved.

The International Medical Device Regulators Forum (IMDRF) has developed a four-tiered framework for categorizing Software as a Medical Device (SaMD) according to the significance of the information the software provides and the seriousness of the healthcare situation in which it is used. Category II, denoting moderate impact on patient and public health, encompasses software that informs clinical management in a critical situation, drives clinical management in a serious situation, or provides information to treat or diagnose in a non-serious situation. Category III, denoting higher impact and the potential for significant harm if improperly managed or used, encompasses software that provides information to treat or diagnose in a serious situation or drives clinical management in a critical situation (15).

Roundtable participants contended that triage, understood here as the making of rapid judgments regarding symptom severity, medical urgency and treatment need, straddles these two mid-level risk categories and firmly fits within this overall framework. With an estimated 230 million people using AI chatbots to self-triage each year (16), there is a compelling case for classifying conversational AI tools as special types of medical devices that require similar levels of safeguarding and governmental oversight as the Category II and III technologies that are more traditionally or intuitively considered “clinical interventions”.

In parallel with their considerations of greatest possible risk, participants also identified lower-risk uses for AI companions when positioned as adjuncts, rather than replacements, for human care. Examples included supporting neurodiverse young adults in interpreting social situations, planning conversations, or organizing daily routines, with clear boundaries around their role in crisis management. Ideally, such tools can be trained to detect health-compromising language from users, such as self-harm ideation, direct patients to more robust resources like hotlines when warranted, and clearly communicate their role as a supplement to clinical care rather than a replacement.

Recognizing that young adults will use AI regardless of clinical endorsement, participants suggested that a harm reduction lens—with the definition of “harm” itself determined in direct collaboration with diverse communities—is the most pragmatic design principle for companion tools targeting this population (17). Applying this lens will require community education on the capabilities and limitations of chatbots, clearer guidance on safer use, and structured pathways for escalation to human support. Each of these priorities offers industry leaders a meaningful opportunity to bring young adult patients into the co-design of AI companion chatbots, not only when shaping the aesthetics of user interfaces, but when determining where and how to communicate terms, conditions, risks, and alternative support options. Finally, participants emphasized the value of peer-led digital literacy initiatives to support young adults' critical and informed use of AI mental health tools in low-pressure, judgment-free settings.

## Conclusion and call to action

Generation Patient's Roundtable on Young Adults with Chronic Medical Conditions offered a timely and necessary examination of the promises and pitfalls of AI in the prevention, diagnosis, treatment, and management of lifelong illness. Bringing together current and former young adult patients, caregivers, healthcare providers, grassroots advocates, institutional representatives, and bioethicists, the convening was centered around a foundational question: can emerging technologies effectively address the health service gaps that human capacity alone cannot fill, without compounding the vulnerabilities of those they purport to serve?

The answer, as reflected in the prevailing sentiment of participants, is that AI has become an inescapable feature of the current cultural and economic landscape. The more urgent task, therefore, is not to debate its prevalence but to shape its trajectory. This requires deliberate effort to identify, minimize, and transparently communicate risk across every stage of AI development and deployment in healthcare settings.

To that end, we call on developers, clinicians, policymakers, health systems leaders, and patient advocates to act with both urgency and intention. Healthcare-oriented AI must be designed to reduce friction in scheduling, insurance navigation, and other help-seeking processes, creating space for more meaningful patient-provider relationships centered on treatment and healing. It must establish secure, interoperable channels for sharing patient histories across pediatric and adult care networks, lifting administrative burden from patients who have already shouldered too much. And it must deliver plain-language, evidence-based guidance that supports self-management and self-advocacy for people navigating chronic illness, with the explicit goal of advancing equitable health outcomes.

Equally, those with the power to build and regulate these tools bear a responsibility to prevent harm. AI must not replace human interaction in ways that foster parasocial dependency or erode the therapeutic relationship. It must not reproduce structural inequities through opaque consent processes, politically motivated data use, or the uncritical incorporation of non-representative training data into high-stakes clinical and administrative decisions. And it must not sacrifice clinical accuracy for increased user engagement, prioritizing palatability over quality of care.

These commitments are not optional features of responsible AI design. They are the baseline. In a sociopolitical climate where access to basic healthcare remains out of reach for far too many, and where people with chronic illness bear disproportionate financial and logistical burdens, AI cannot be permitted to function as a substitute for equitable care. Regulatory oversight and clear risk communication must advance in parallel with patient advocacy that makes human-centered treatment safer, more accessible, and more responsive to the needs of those most often left behind.

This is the vision at the foundation of Design Justice, and it is the standard to which AI innovation in healthcare must be held. The path forward requires ongoing, democratic dialogue among all stakeholders, from patients to providers, and individual developers to large technology corporations, who share both the privilege of shaping these tools and the responsibility of ensuring they serve everyone. That dialogue must begin now, and it must begin with patients at the forefront.

## Data Availability

The original contributions presented in the study are included in the article/Supplementary Material, further inquiries can be directed to the corresponding author.
